# A Case of Nipple Adenoma With Long-Term Follow-Up Without Change in Malignancy

**DOI:** 10.7759/cureus.68578

**Published:** 2024-09-03

**Authors:** Kenji Yorita, Norihiro Hokimoto

**Affiliations:** 1 Diagnostic Pathology, Japanese Red Cross Kochi Hospital, Kochi, JPN; 2 Surgery, Japanese Red Cross Kochi Hospital, Kochi, JPN

**Keywords:** follow up, pathology, surgery, breast, nipple adenoma

## Abstract

Nipple adenomas are rare benign tumors. The risk of carcinogenesis and the exact time of cancerization of nipple adenomas is unknown. We report a case of nipple adenoma in a 76-year-old Japanese woman. At the age of 52, she noticed erosion of her left nipple and was diagnosed with nipple adenoma by biopsy at a nearby hospital. A follow-up of the left nipple lesion was performed at the patient’s request; however, she refused to undergo mammography and biopsy. The patient requested surgery for the left nipple lesion after 24 years of follow-up because of increasing bloody exudates from the nipple lesion. The preoperative radiological examination did not reveal malignancy in either breast, and a left nipple-areolar resection was performed. A nipple adenoma was identified by pathological diagnosis; however, no malignant findings were observed. The present case appears to have the longest follow-up period ever reported. This case is valuable because follow-up may be a clinical management option for nipple adenomas.

## Introduction

Nipple adenoma is a rare benign tumor [1]. Surgical management for nipple adenomas is often selected due to their troublesome symptoms, as supported by numerous published surgical reports [2]. Follow-up studies for nipple adenomas have been reported, and changes in malignancy of nipple adenomas are believed to be rare [2,3]. Several cases of nipple adenomas becoming cancerous over a long period have been reported [3-6]. Therefore, a long-term follow-up for nipple adenomas might be important to learn about their natural history. However, the natural history of nipple adenomas may not be well known as long-term follow-up studies, as was seen in our case, have been hardly reported. To the best of our knowledge, the longest follow-up period for nipple adenoma was approximately 19 years in previous cases [3]. Here, we report the case of a 76-year-old Japanese woman with a left nipple adenoma with 24 years of follow-up. Such long-term follow-up has not yet been reported. After follow-up, surgery for nipple adenoma was performed as per her request, and a pathological evaluation was conducted. Clinicopathological findings in our case are presented, and the management of the nipple adenoma is discussed.

## Case presentation

A 68-year-old Japanese woman was referred to our hospital for a follow-up of a left nipple lesion caused by movement. She noticed the erosion of the left nipple at the age of 52, and nipple adenoma was diagnosed by biopsy at another hospital (detailed data were not available), where the nipple lesion had been followed up as requested. Other medical histories included type 2 diabetes mellitus and hypertension. No family history of breast or ovarian cancer was reported.

**Figure 1:**
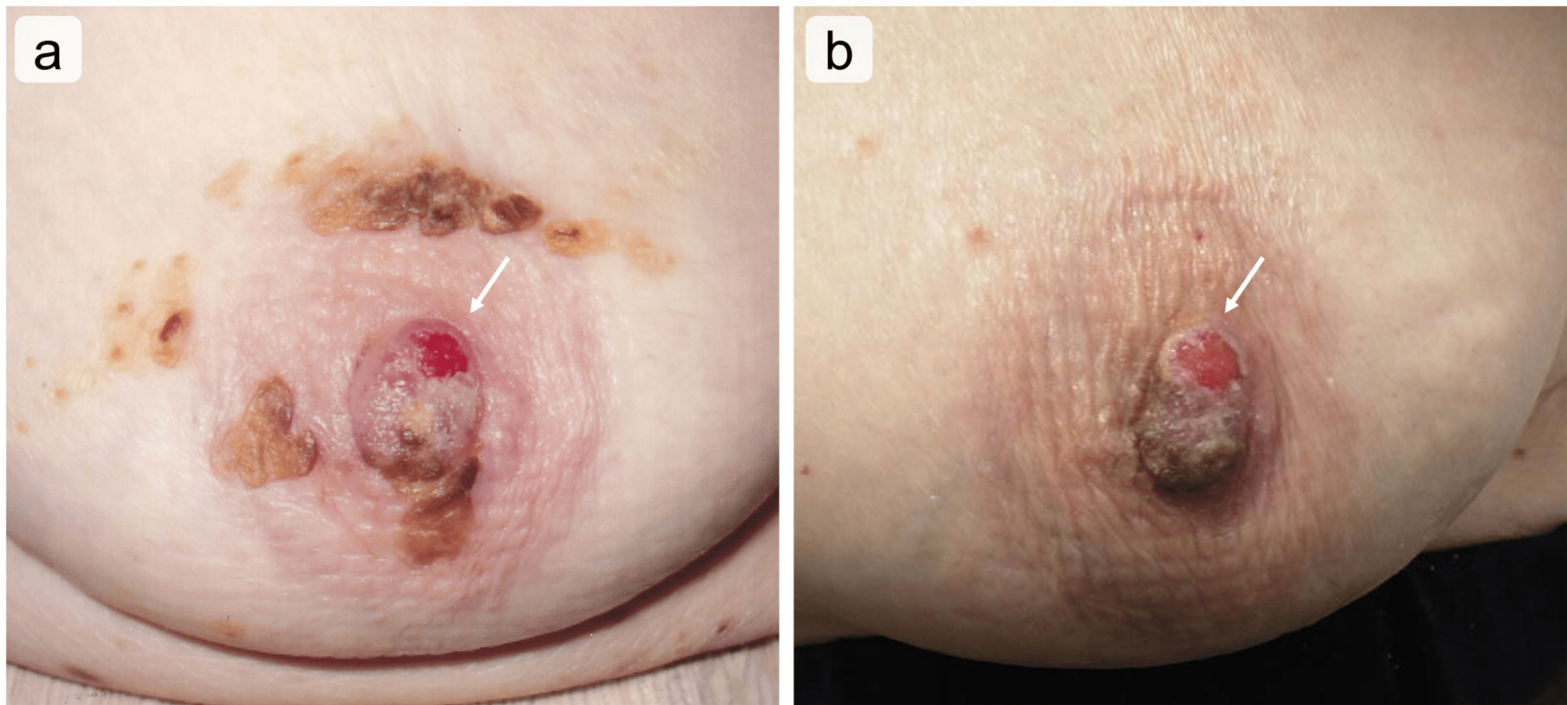
Gross appearance of the nipple adenoma of the left breast The photos were taken (a) eight years ago and (b) during the preoperative state. The eroded portion of the left nipple (arrows) is seen at the upper and outer nipple portion in (a) and in the upper, inner, and outer nipple portion in (b).

The nipple lesion appeared as an erosion on the upper and outer portions of the left nipple at age 68 (Figure 1a). Routine mammography every 1-2 years was recommended; however, the patient refused. The incidence of hemorrhagic exudates from the nipple lesion increased over the past several years. A second biopsy of the nipple lesion was recommended, but the patient declined. As the increasing exudate from the lesion began to affect her, she requested surgery for the nipple lesion at 76 years. Preoperatively, the left nipple lesion (Figure 1b) appeared to be slightly enlarged owing to the upper inner and outer localization of the nipple compared to the lesion at age 68 (Figure 1a).

**Figure 2:**
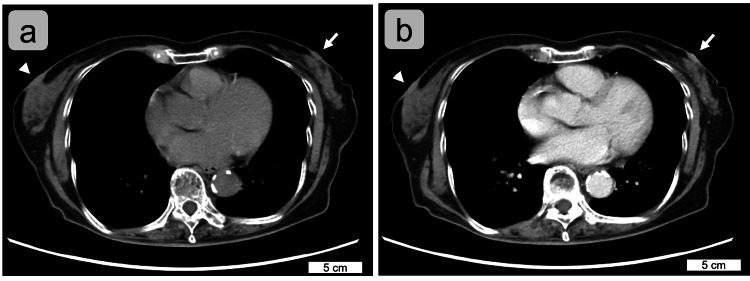
Preoperatively-taken computed tomographic images of the nipple adenoma (a) Non-enhanced and (b) contrast-enhanced images. The left nipple lesion (arrows) may be slightly enhanced compared to the right nipple (arrowheads). Scale bars are shown in the images.

Preoperative mammography and contrast-enhanced computed tomography (Figure 2) revealed no abnormalities.

Nipple-areolar resection of the nipple lesion and pathological examination were performed.

**Figure 3:**
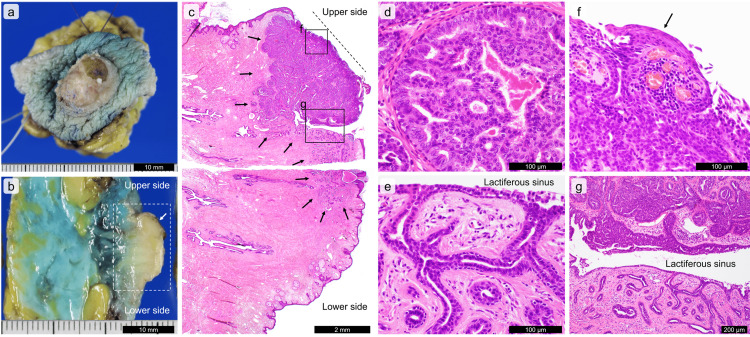
Macroscopic and microscopic images of the nipple adenoma (a-b) A formalin-fixed resected tissue. A cut surface of the nipple adenoma (b) corresponds to the dotted line in (a). A vague nodule (an arrow) is seen in the eroded portion in (b). (c-g) Microscopic images of the nipple adenoma with hematoxylin and eosin-stained section. A low-magnified photo (c) corresponds to the dotted quadrangle in (b). A dotted line in (c) corresponds to the erosive lesion. The nipple adenoma consists of plump cuboidal cells arranged in cribriform-like (d) and irregular glandular (e) nests. The tumor cells involve the epidermis (f) (an arrow) and a lactiferous duct (g). Scale bars are shown in the images.

Macroscopically, the eroded nipple lesion appeared as a solid nodule on the cut surface (Figure 3a, Figure 3b). A whole-mount preparation of the resected tissue was performed. Histologically, a left nipple lesion was observed in the grossly eroded portion as well as in the non-eroded portion, with a size of 7×4 mm (Figure 3c). The nipple lesion consisted of cuboidal cells with round or oval nuclei, forming tubules or cribriform-like nests and preserving a two-layered structure. Mitosis was not seen. The grossly eroded portion mainly consisted of cribriform-like nests (Figure 3d), and the non-eroded portion mainly consisted of irregular tubules (Figure 3e). In the grossly eroded portion, tumor cells were involved and replaced the epidermis (Figure 3f). Cribriform-like and tubular epithelia were present in the epithelium of the lactiferous sinus (Figure 3e, Figure 3g). The papillary luminal epithelium was hardly seen. Apocrine metaplasia of the glandular epithelium was observed in the grossly non-eroded portion. Paget disease was absent. No neoplasia was observed in the breast parenchyma.

**Figure 4:**
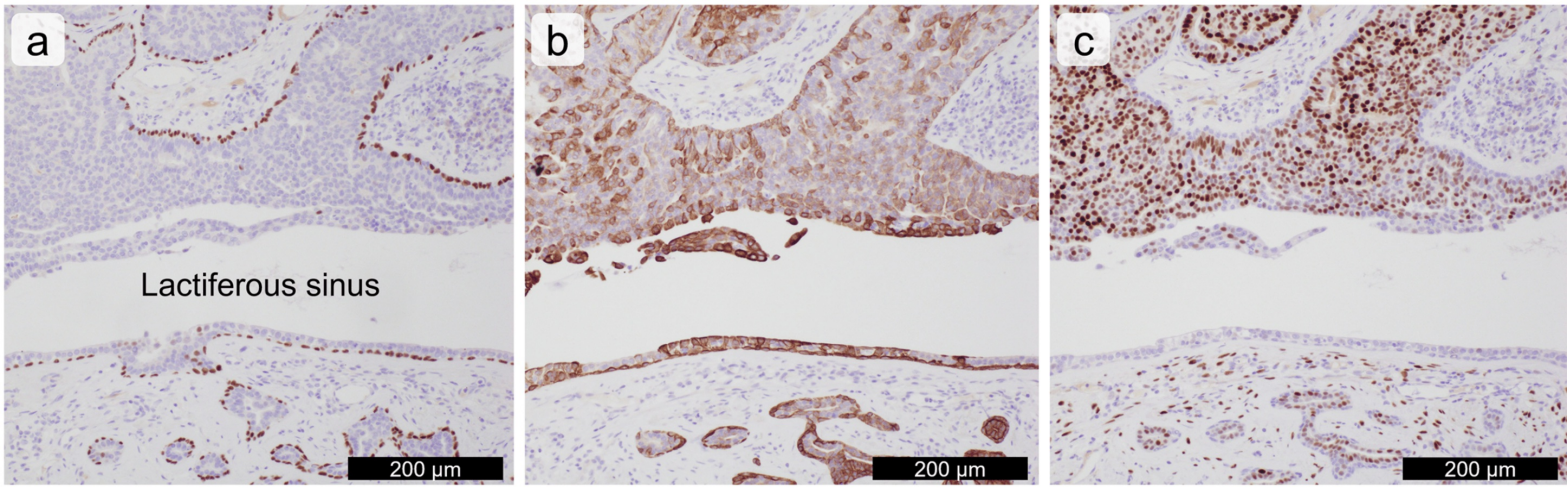
Immunohistochemical images of the nipple adenoma (a) p63, (b) cytokeratin 5, and (c) estrogen receptor. Scale bars are shown in the images.

Nipple adenoma was mostly considered in hematoxylin and eosin-stained sections. However, ductal carcinoma in situ (DCIS) could not be ruled out in grossly eroded areas. Immunohistochemically, the tubular and cribriform-like nests completely preserved the two-layered structure owing to the presence of p63-positive myoepithelial cells (Figure 4a). The luminal epithelium of the tubular and cribriform-like nests showed a mosaic positive pattern of cytokeratin 5 (Figure 4b), largely positive for the estrogen receptor (Figure 4c), and almost negative for the progesterone receptor. Thus, DCIS was denied. In total, nipple adenoma with usual ductal hyperplasia was considered.

The patient was discharged from the hospital without any complications and followed up for two weeks after the operation. The patient provided informed consent to participate in this study.

## Discussion

We present a case of nipple adenoma with a long-term follow-up of 24 years.

Nipple adenoma is rare (0.0125%, 38 cases/305000 surgical specimens [1]), with an age at diagnosis ranging from two years [7] to 83 years [8]. Nipple adenomas were not associated with a family history of breast carcinoma [4]. Symptoms of nipple adenoma, including pain, itching, exudation, and bleeding, appear to be induced by tumor cells causing nipple erosion. Nipple adenomas create ill-defined or nodular lesions, as observed in our case. In our case, the nodular component corresponded to the usual ductal hyperplasia of the ductal epithelium.

Other histological differential diagnoses include syringomatous tumors and syringocystadenoma papilliferum. Syringomatous tumors were ruled out because they did not involve the surface squamous epithelium or lactiferous sinus and usually tested negative for hormone receptors [9]. Syringocystadenoma papilliferum was unlikely because of the lack of papillary proliferation in the squamous epithelium. Nipple adenomas are also known as florid papillomatosis [4]. Florid glandular proliferation was observed in these patients. We used nipple adenoma rather than florid papillomatosis because the nipple lesion in our patient hardly showed papillary growth, and the recent World Health Organization classification of breast tumors does not recommend the use of florid papillomatosis [10].

Patients may have nipple adenoma and breast carcinoma, which appear metachronously or synchronously in their lives or in spatially separate locations. Nipple adenoma and breast carcinoma are generally believed to be coincidental [4,11]. In 967 breast cancer patients, nipple adenomas were histologically and incidentally detected in 12 cases (1.2%) who underwent mastectomy [12]. If a nipple adenoma is clinically detected, a careful breast examination should be performed to rule out the presence of coincidental breast carcinoma.

Surgical management for nipple adenoma has been well-reported. In this case, partial nipple excision using nipple-preserving techniques (wedge resection, enucleation, and Mohs micrographic surgery) could have been selected because the nipple adenoma was a benign lesion confined to the nipple. However, nipple-areolar resection was performed because the malignant transformation of the lesion could not be ruled out preoperatively, owing to the long-term course of the disease. A long-term follow-up study to clarify the natural history of nipple adenomas appears to be rare [2]. To the best of our knowledge, the longest follow-up period for nipple adenoma was 227 months (approximately 19 years) in previous cases [3], and our patient has the longest follow-up period. Cases of malignant changes in nipple adenoma are rare [2], and the textbook of Rosen’s Breast Pathology describes 13 cases of nipple adenoma with malignant changes [4]. Nipple adenoma may be a precancerous condition in men but is less substantial in women [4]. The histology of malignancies derived from nipple adenomas has been reported as follows: DCIS [13], invasive breast carcinoma [3,5,6,14], and low-grade adenosquamous carcinoma [15]. Although the precise duration from the diagnosis of nipple adenoma to the diagnosis of malignant changes has not been evaluated, the following cases have been reported. Among 44 cases of nipple adenoma, only one case showed that invasive breast carcinoma was detected adjacent to the excision site at 98 months (approximately eight years) of follow-up [3]. Abdulwaasey et al. reported invasive breast carcinoma arising from a nipple adenoma 15 years after diagnosis [5]. Rao et al. reported that a male nipple adenoma with DCIS progressed to invasive breast cancer nine years after diagnosis [6]. Thus, the long-term follow-up of nipple adenomas may be important.

## Conclusions

A Japanese woman with no past or family history of breast cancer followed up for 24 years with a nipple adenoma at her request. To the best of our knowledge, this is the longest follow-up case reported to date. Although nipple adenoma can be an incidental complication of breast cancer and should be treated with caution because it can rarely become cancerous, there was no development of breast cancer or carcinomatosis of the nipple adenoma during follow-up. In addition to surgical treatment, long-term follow-up may be considered an option for the clinical management of nipple adenomas.
